# Merkel Cell Carcinoma of the Left Eyelid with Metastasis to the Left Submandibular Lymph Nodes: A Case Report and Brief Review

**DOI:** 10.1155/2022/4712301

**Published:** 2022-02-24

**Authors:** Caroline Casey, Nina Gallo, Samuel Gallo

**Affiliations:** ^1^University of Central Florida College of Medicine, Orlando, FL, USA; ^2^Gallo Eye and Facial Plastic Surgery, Dublin, OH, USA

## Abstract

Merkel cell carcinoma (MCC) is a cutaneous cancer often found on sun-exposed areas. MCC is rare but very often lethal making early diagnosis challenging although critical. There are only a few cases that have reported MCC of the eyelid making it often hard to identify in clinic. A 52-year-old woman with a firm nodule on the left eyelid was diagnosed with MCC that had also metastasized to a left submandibular lymph node. She underwent surgical excision of the mass and lymph node as well as parotid gland and neck dissection to rule out other metastases and then underwent radiation therapy. The aim of this study is to report a case of eyelid MCC with metastasis to a local lymph node to provide another example of the rare cancer in hopes that diagnosis and treatment options for MCC are more thoroughly studied and understood.

## 1. Background

Merkel cell carcinoma (MCC) is a rare but aggressive skin cancer, with around 1500 new cases in the United States per year and a mortality rate of up to 40% [1]. It is a cancer with neuroendocrine features that was first described as a “trabecular carcinoma of the skin” in 1972; however, the cancer cell of origin is still debated [2–5]. MCC is associated with either Merkel cell polyomavirus or chronic ultraviolet (UV) light exposure, both of which cause DNA mutations that are characteristic for the condition [1]. Most cases of MCC are found as painless, expanding lesions on sun-exposed areas, and it is more commonly found in Caucasians, males, and the elderly [1]. Immunosuppression is another key risk factor that is more commonly associated with MCC found in younger patients [1]. Eyelid MCC tumors make up around 2.5% of all cases, and women are around two times more likely to have eyelid MCC compared to men [1]. MCC on eyelids is more commonly found on the upper eyelid; this is also where Merkel cells in healthy eyelids are typically found [1, 6, 7]. MCC of the eyelid is often misdiagnosed as cysts, chalazion, or basal cell carcinomas as it is a rare tumor; therefore, an accurate diagnosis is often delayed [1].

## 2. Case Presentation

A 52-year-old white female presented upon referral in June of 2013 with a recurrent lesion of the left eyelid that was previously diagnosed as a chalazion. Her past medical history was significant for hypertension, measles, mumps, scarlet fever, ruptured lumbar disk, and arthritis, among a few other conditions. She had a family history of stomach cancer with metastases to the rectum in her mother; prostate cancer in her father; breast cancer in her sister, maternal aunt, and cousin; and pancreatic cancer in her grandfather.

**Figure 1:**
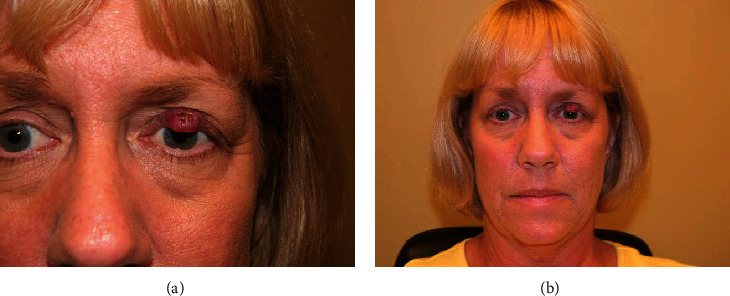
Merkel cell carcinoma (MCC) of the left eyelid in this patient. (a) Closeup image of tumor and (b) head-shot of the patient depicting the size of the tumor in comparison to facial structures.

Her ophthalmic history was unremarkable, and she had no reports of growths in the past aside from what was believed to be a recurrent chalazion. The lesion was initially excised by her general ophthalmologist. No pathology was sent. Upon recurrence, the patient was sent for a second opinion. The lesion (Figure 1) was biopsied, and histopathology was performed. A malignant neoplasm was found extending to the base of the biopsied specimen. The diagnosis was found to be MCC.

Positron emission tomography (PET) and computed tomography (CT) scans revealed an enlarged left submandibular lymph node measuring 1.8 × 1.4 cm with a standardized uptake value (SUV) of 8.5. A fine needle aspiration of the lymph node revealed neuroendocrine carcinoma indicating metastatic MCC.

A wide excision of the tumor and the left submandibular neck contents was completed in July, and one out of two lymph nodes was positive for metastatic MCC. In August, a further neck dissection was completed. The skin excision site was negative for carcinoma. The left lateral parotid lobe was excised, and the salivary gland was found to be without abnormalities. A left cervical lymphadenectomy was also performed. Level I neck contents along with left neck contents in levels II-IV were negative for metastases. All lymph nodes were negative for metastatic carcinoma in levels II-V.

Left eyelid radiation (50.4Gy) was started in October and definitively completed in November of 2013. There has been no evidence of disease since 2013. The patient has since been counseled on UV light protection and self-checks of nevi and other suspicious skin lesions. She has attended regular follow-ups with labs, CXR, and exams of nevi that revealed no evidence of disease.

In 2014, the patient had biopsy-confirmed basal cell carcinoma (BCC) removed from her right upper arm and back. Both lesions were fully excised. In 2014, the patient also had shingles in her right temple/forehead.

Since those incidents, the patient has had no significant skin findings or concerns and is being followed biannually.

## 3. Discussion

MCC is an aggressive cancer with a high rate of metastases and a high mortality rate, and the number of cases has been on the rise over the past few years [8]. Lesions often first appear as red, firm nodules with overlying telangiectasias possible; however, 5% of MCC are found in the lymph nodes without any skin findings [8, 9]. Diagnosis of MCC is via biopsy, which can reveal small, round, blue cells with a high nucleus to cytoplasm ratio, many mitoses, and dense granules found in the cytoplasm [10]. After diagnosis, appropriate staging is necessary in order to develop a treatment course and effectively counsel patients [8]. With a high propensity to metastasize, full-body PET scans have been found to be most effective at evaluating for distant metastases [8].

MCC has a high rate of local recurrence; so, early diagnosis, rapid treatment, and consistent follow-ups are important to ensure disease remission [11]. Additionally, MCC has a high rate of early metastases to regional lymph nodes, and metastases are more common with a tumor size greater than 2 cm [11]. It is rare for MCC to be found in the area of the eye or eyelid—there are only around 200 reported cases [1]. However, when it is seen, it is often misdiagnosed, as it was initially in this case. There have been previous reports of metastases from the eyelid to regional lymph nodes, parotid lymph nodes, preauricular nodes, submandibular nodes, and distant sites with larger tumors—such as the lungs [12, 13]. However, MCC of the eyelid/eye still remains a very rare finding.

Surgical resection is the mainstay of treatment for localized MCC tumors and with a high rate of recurrence, and immediate excision with clear margins is indicated [14]. Management of metastatic disease; however, involves a multidisciplinary approach. Radiation therapy has been long debated, but is used for MCC involving the lymph nodes or where there is extrascapular extension and has been shown, when combined with surgery, to reduce the risk of local recurrence [14]. Hyperthermic isolated limb perfusion, where high doses of chemotherapy are injected under hyperthermic conditions, is another method that has been shown to provide some clinical benefit [14]. Recently, an immunotherapy agent, Avelumab, in clinical trials has shown some efficacy in treating chemotherapy-resistant MCC [15]. The JAVELIN trial has shown positive results for the treatment of metastatic MCC, and Avelumab is FDA-approved as of June 2020 for treatment of disseminated MCC [16]. MCC is an aggressive cancer, and there continues to be development of treatment options for metastatic disease.

## 4. Conclusion

In conclusion, this is one of few reports documenting MCC of the eyelid with metastasis to the left submandibular lymph node. Given MCC's high mortality rate, risk of recurrence, and propensity to metastasize, documenting these cases remains critical as treatment strategies and early diagnostic strategies are needed.
